# Extracts from Glioma Tissues following Cryoablation Have Proapoptosis, Antiproliferation, and Anti-Invasion Effects on Glioma Cells

**DOI:** 10.1155/2014/236939

**Published:** 2014-04-10

**Authors:** Tianzhu Liu, Xin Wang, Zhilin Yin, Jun Pan, Hongbo Guo, Shizhong Zhang

**Affiliations:** Department of Neurosurgery, Neurosurgery Institute of Guangdong Province, Key Laboratory on Brain Function Rebuild and Rehabilitation of Guangdong, Zhujiang Hospital, Southern Medical University, Guangzhou 510282, China

## Abstract

*Objective*. This study is to investigate the *in vivo* apoptotic processes in glioma tissues following cryoablation and the effects of glioma tissue extracts on GL261 glioma cells *in vitro*. *Methods*. TUNEL and flow cytometry analysis were performed to detect the apoptotic processes in the glioma tissues following cryoablation and in the GL261 cells treated with cryoablated tumor extracts. The scratch assay, the transwell assay, and Western blot analysis were carried out to evaluate the effects of cryoablated tumor extracts on the migration, invasion, and proliferation of tumor cells. *Results*. Our *in vivo* results indicated that the rapid-onset apoptosis was induced via the intrinsic pathway and the delayed apoptosis was triggered through the extrinsic pathway. The *in vitro* results showed that extracts from glioma tissues following cryoablation induced apoptosis via extrinsic pathways in GL261 glioma cells. Furthermore, cryoablated tumor extracts significantly inhibited the migration and proliferation of these cells, which would be related to the inhibition of ERK1/2 pathway and the activation of P38 pathway. *Conclusion*. Glioma cells surviving in cryoablation undergo intrinsic or extrinsic apoptosis. Augmenting the induction of apoptosis or enhancing the cryosensitization of tumor cells by coupling cryoablation with specific chemotherapy effectively increases the efficiency of this therapeutic treatment.

## 1. Introduction

Gliomas are the most common tumors in the central nervous system of adults, accounting for approximately 80% of the malignant primary brain tumors [1]. Chemotherapy and radiation therapy are the most common treatments today for the patients with glioma. However, the prognosis is generally poor, with an average survival time of 51 weeks [2].

In recent years, cryoablation of pathological tissues has become an increasingly popular method of treatment for a variety of tumors, including skin, oral cavity, liver, bone, cervical, prostate, and brain cancers [3, 4]. Cryoablation is a minimally invasive surgical technique, established as rapid freezing, slow thawing, and repetition of the freezing/thawing cycles [5]. There are two major well-known mechanisms through which cryoablation induces cellular damage, death, and necrosis of tissues, that is, the direct rupture of cells caused by intra/extracellular ice crystal formation and the microcirculatory failure which occurs in the thawing period [5]. Furthermore, increasing evidence indicates that apoptosis, or programmed cell death, would be a major contributor to cell death after cryosurgery [6–8]. Following cryoablation, damaged or ruptured cells would release cellular contents, which contain numerous apoptosis-related signaling factors, including tumor necrosis factor *α* [9], reactive oxygen species [10], proteinases, and high concentration of potassium ions [11]. Moreover, extracellular adenosine triphosphate (ATP) released by the injured and/or destroyed cells has also been shown to induce apoptosis via stimulating P2X7 receptors [12, 13]. However, the mechanism of cryoinduced apoptosis and its relationship with the released cellular contents have not yet been established.

In this study, the apoptotic process in glioma tissues following cryoablation was detected* in vivo*, and the effects of the cryoablated glioma tissue extracts on GL261 glioma cells were also investigated* in vitro*. Our results demonstrate that cryoablation induces two waves of apoptosis in glioma tissues, and the extracts from glioma tissues following cryoablation exert proapoptosis, antiproliferation, and anti-invasion effects on GL261 glioma cells.

## 2. Materials and Methods

### 2.1. Cell Culture

The murine glioma cell line GL261 was purchased from American Type Culture Collection and cultured in Dulbecco's modified Eagle's medium (DMEM; Hyclone, Logan, Utah, USA), supplemented with 10% (v/v) fetal bovine serum (FBS; Gibco, Grand Island, NY, USA), 2 mM glutamine, as well as 100 units/mL penicillin, and 100 mg/mL streptomycin (Hyclone). Cells were incubated at 37°C with 95% air and 5% CO_2_.

### 2.2. Experimental Animals, Freezing Procedure, and Preparation of the Cryoablated Tumor Extracts

C57 mice of either gender (*n* = 50, 4–6 weeks old) were obtained from the Southern Medical University Animal Center (Guangzhou, China). Mice were maintained under standard laboratory conditions (12 h light-dark cycle, lights on from 7:00 a.m. to 7:00 p.m.), with free access to food and water. All of the experiments were conducted in accordance with the Regulations for the Administration of Affairs Concerning Experimental Animals (China) and were approved by the Southern Medical University Animal Ethics Committee.

**Figure 1:**
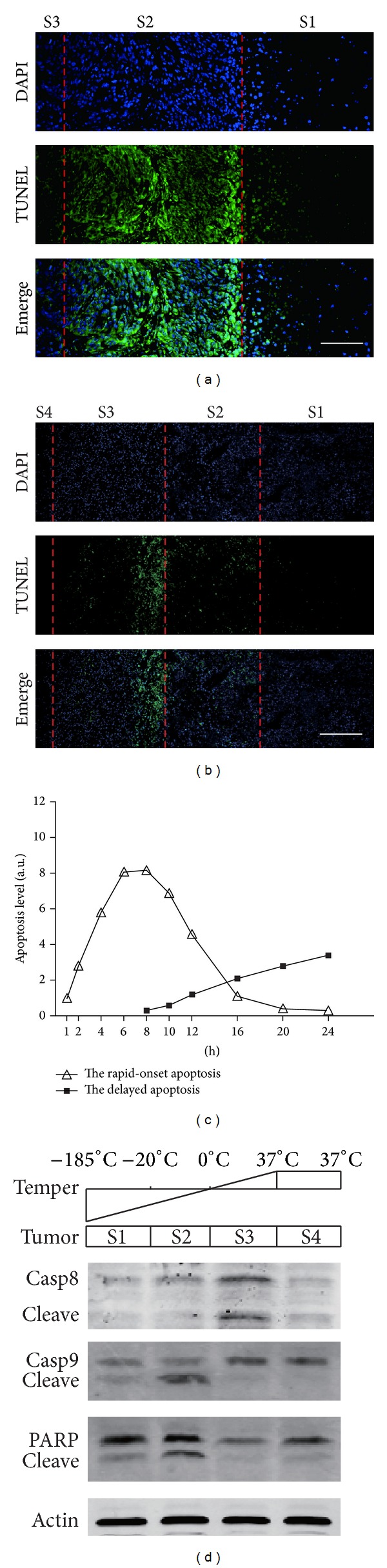
Apoptosis analysis in glioma tissues following cryoablation. (a) Apoptosis in glioma tissues at 8 h after cryoablation for three cycles of rapid freezing/thawing detected via the TUNEL assay. Scale bar, 50 *μ*m. (b) Apoptosis in glioma tissues at 24 h after cryoablation for one cycle of rapid freezing/thawing detected via the TUNEL assay. Scale bar, 100 *μ*m. (c) The comparison of the levels of the rapid-onset apoptosis and the delayed apoptosis in glioma tissues at different time points after cryoablation for one cycle of rapid freezing/thawing. (d) The levels of PARP, procaspase-8, procaspase-9, and their cleaved forms in the four sections of tumor tissues at 24 h after cryoablation for one cycle of rapid freezing/thawing were analyzed via Western blot. S1–S4, Sections 1–4, in a tumor with the treatment of cryoablation: S1, the center of frozen tissues, S2, peripheral region of frozen tissues, S3, areas outside the frozen tissues, and S4, normal tissues of mice surrounding the tumor.

GL261 glioma cells (approximately 1 × 10^7^ cells) were subcutaneously inoculated in the dorsal region of the mouse, bilaterally. When tumors were grown to 15–20 mm in diameter, the tissues were subjected to cryoablation. These tumor-bearing mice were randomly assigned to two groups: (1) the group treated with cryoablation and (2) the sham-operated control group. The Cryocare Cryosurgical System (Endocare, Irvine, CA, USA) was used for cryoablation. Mice were anesthetized with isoflurane during the entire surgical procedure. After an incision was made in the tumor, the cryoprobe (3 mm) was placed in contact with it for one or three cycles of rapid freezing/thawing (20/40 s each). Sham operation was done with the cryoprobe placed into the tumor at the same depth without cryoablation. Tumor samples were dissected from mice at 1, 2, 4, 6, 8, 10, 12, 16, 20, and 24 h after cryoablation and then subjected to protein extraction and paraffin embedding, respectively. The tumor samples at 24 h after cryoablation were divided into 4 sections, as described in Figure 1(d), and immediately frozen in liquid nitrogen.

Tumors dissected from mice at 2 h after cryoablation under sterile conditions were used to prepare cryoablated tumor extracts. Briefly, cryoablated tumor tissues around the cryoprobe were separated, minced, and pipetted gently in DMEM (1 g tumor tissues in 1 mL DMEM). After 30 min, the suspension was centrifuged at 5000 r/min for 10 min and then filtrated to remove tissues pieces. The remaining solution, regarded as cryoablated tumor extracts, was used for the following* in vitro* experiments. Extracts from the sham-operated tumors via the same procedures were used as control.

### 2.3. Apoptosis Analysis

Apoptosis in glioma tissues after cryoablation and in GL261 glioma cells treated with cryoablated tumor extracts (1 mL extract solution/1 mL DMEM) was investigated by the terminal deoxynucleotidyl transferase dUTP nick end labelling (TUNEL) assay (Roche, Indianapolis, IN, USA), according to the manufacturer's instructions. For the assessment of the cells with blockages of caspase-8, caspase-9, and P2X7, these cells were treated with cryoablated tumor extracts, together with 10 *μ*M IETD (Biovision, Wehrheim, Germany), 10 *μ*M LEDH (Biovision), and 10 *μ*M A740003 (Sigma, St. Louis, MO, USA), respectively, for 12 h. Then they were subjected to the TUNEL assay.

Apoptosis in GL261 glioma cells treated with cryoablated tumor extracts was further observed by flow cytometry analysis. After cryoablated tumor extract treatment (1 mL extract solution/1 mL DMEM) for 12 h, cells were washed with cold PBS and resuspended with binding buffer (10 mM HEPES NaOH, pH 7.4, 140 mM NaCl, 2.5 mM CaCl_2_). Cells were stained for 15 min at room temperature in the dark with annexin V-FITC and propidium iodide and then analyzed by a Becton Dickinson FACScan (excitation light at 488 nm) equipped with the CELLQUEST software (Becton Dickinson, Mountain View, CA, USA).

### 2.4. Western Blot

Western blot was performed as described previously [14]. Primary antibodies were as follows: rabbit monoclonal anti-p-ERK1/2 antibody (1 : 500; Cell Signaling Technology, Inc., Beverly, MA, USA), mouse polyclonal anti-ERK1/2 antibody (1 : 150; Santa Cruz Biotechnology, Inc., Santa Cruz, CA, USA), mouse monoclonal anti-p-JNK antibody (1 : 500; Cell Signaling Technology, Inc.), rabbit polyclonal anti-JNK antibody (1 : 150; Santa Cruz Biotechnology, Inc.), rabbit polyclonal anti-p-P38 antibody (1 : 500; Cell Signaling Technology, Inc.), rabbit polyclonal anti-P38 antibody (1 : 150; Santa Cruz Biotechnology, Inc.), goat polyclonal anti-Ki-67 antibody (1 : 150; Santa Cruz Biotechnology, Inc.), mouse monoclonal anti-caspase-8 antibody (1 : 100; Santa Cruz Biotechnology, Inc.), rabbit polyclonal anti-caspase-9 antibody (1 : 200; Santa Cruz Biotechnology, Inc.), mouse monoclonal anti-PARP antibody (1 : 150; Santa Cruz Biotechnology, Inc.), and mouse monoclonal anti-*β*-actin antibody (1 : 150; Santa Cruz Biotechnology, Inc.). Secondary antibodies were as follows: donkey anti-goat, goat anti-mouse, and goat anti-rabbit IRDye 800CW secondary antibodies (1 : 15000; Li-Cor Biosciences, Lincoln, NE, USA). Quantification was performed using an Odyssey infrared imaging system (Li-Cor Biosciences).

### 2.5. Migration and Invasion Assays

The migration of GL261 cells was determined by the scratch assay. Cells were seeded into six-well tissue culture plates. When the cells reached 90% confluence, a straight scratch wound was created by gently removing the attached cells using a 100 *μ*L standard pipette tip. The wounded monolayer cells were washed with fresh medium once to remove floating cells, and then fresh mixed medium (1 mL extract solution/1 mL DMEM) supplemented with 10% FBS was added. After incubation for 12 h, cells that migrated across the marked reference line were photographed. The migrated distances were calculated by subtracting the distance between the lesion edges at 12 h from the initial distance. Five areas were selected randomly from each well, and cells in three wells of each group were quantified in each experiment.

Furthermore, invasion assay was conducted in a 24-well plate with Matrigel-coated transwell filters (8 *μ*m pore size; Millipore, Bedford, MA, USA). Cells (approximately 1 × 10^5^) were suspended in serum-free medium containing cryoablated tumor extracts (1 mL exact solution/1 mL DMEM) and then loaded in the top chamber. Medium with 10% FBS was placed in the lower chamber as chemotactic stimulus. After incubation for 12 h, the nonmigrated cells on the top chamber were removed with a cotton swab. The migrated cells on the bottom of the chamber were fixed with 4% paraformaldehyde and stained with crystal violet. The migrated cells were photographed and quantified by inverted fluorescent microscope (Leica, Bensheim, Germany). The stained cells were counted from ten randomly selected fields per well. In all experiments, data were collected from triplicate chambers.

### 2.6. Proliferation Assay

Cell counting kit-8 (CCK-8; Dojindo Molecular Technologies, Dojindo, Japan) assay was used to determine the proliferation of GL261 cells. Cells were first dissociated into single cells by trypsin and seeded into 96-well plate (1 × 10^4^ cells per well). 100 *μ*L mixed medium (45 *μ*L extract solution + 45 *μ*L DMEM + 10 *μ*L FBS) was added into each well. Medium was changed every day, until cells were ready for the CCK-8 assay. 10 *μ*L CCK-8 solution was added into each well. After incubation at 37°C for 2 h in a humidified CO_2_ incubator, absorbance was read at 450 nm on a microplate reader (Thermo Varioskan Flash, Thermo, USA). All assays were carried out independently in triplicate.

### 2.7. Statistical Analysis

Data were expressed as means ± standard deviation (SD). Results were analyzed using SPSS13.0 software (SPSS Inc., Chicago, TL, USA). Comparison between experimental groups was made by Student's *t*-test. A value of *P* < 0.05 was considered statistically significant.

## 3. Results

### 3.1. Two Waves of Apoptosis Are Induced in Glioma Tissues following Cryoablation

To detect the apoptosis in glioma tumor tissues after cryoablation, TUNEL staining was performed on the tumor slices. In this assay, the tumor tissue slices were divided into four sections, Sections 1–4 (S1–S4), based on the degree of cryoinjury. In the tumor tissues at 8 h after cryoablation, the results from TUNEL staining showed homogeneous necrosis in S1 (the center of frozen tissues), a wave of apoptosis in S2 (peripheral region of frozen tissues), and no detectable changes in S3 (areas outside the frozen tissues) (Figure 1(a)). In the tumor tissues at 24 h after cryoablation, we detected homogeneous necrosis in S1, a reduced level of apoptosis in S2, and, interestingly, a delayed but strong wave of apoptosis in S3 (Figure 1(b)). Given that the apoptotic process may last for hours, these two waves of apoptosis might be distinguished in a wide time frame. In order to test this, the apoptosis in the tumor tissues was analyzed at 1, 2, 4, 6, 8, 10, 12, 16, 20, and 24 h, respectively, after cryoablation. Our results revealed rapid-onset apoptosis in S2, which started at 1-2 h, peaked at 4–10 h, and declined at 16 h, after cryoablation; the delayed apoptosis was detected in S3, which started at 8 h and peaked at 16–24 h after cryoablation (Figure 1(c)). These data suggest that rapid-onset apoptosis occurs in S2 in the tumor tissues following cryoablation, which might be directly induced by low temperature, while there exists a delayed wave of apoptosis in S3, whose mechanism is unknown.

### 3.2. The Rapid-Onset Apoptosis Is Induced via Intrinsic Pathway While the Delayed Apoptosis Is Triggered by Extrinsic Pathway

We next tried to investigate whether the extrinsic or the intrinsic pathway was responsible for the delayed apoptosis. Therefore, we employed Western blot to assess the expression levels of apoptotic proteins involved in the extrinsic (caspase-8) and the intrinsic (caspase-9 and PARP) pathways, in the tumor tissues at 24 h after cryoablation. Our results showed that the levels of procaspase-9 and PARP were decreased, while the levels of cleaved caspase-9 and PARP were elevated, in S2, indicating that the proteins were cleaved to be activated. However, in S3, the expression levels of procaspase-8 and its cleaved form were increased (Figure 1(d)). These data indicate that the rapid-onset apoptosis in S2 is induced via the intrinsic pathway, while the delayed apoptosis in S3 is triggered by the extrinsic pathway.

### 3.3. Extracts from Glioma Tissues following Cryoablation Induce Apoptosis via Extrinsic Pathway in GL261 Glioma Cells

**Figure 2:**
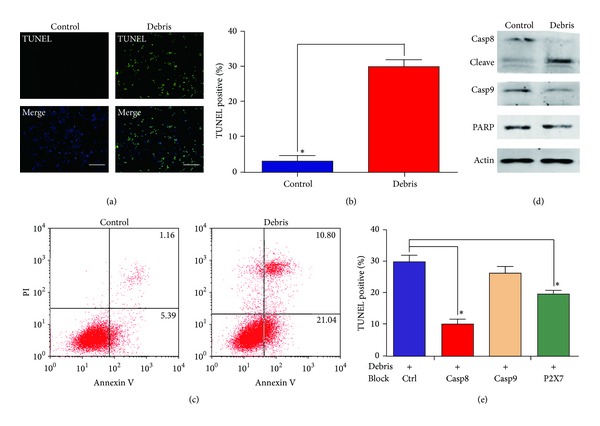
Cryoablated tumor extracts induced GL261 glioma cell apoptosis via extrinsic pathway. (a) TUNEL assay detecting the apoptotic processes in the GL261 cells. GL261 cells were treated with cryoablated tumor extracts (1 mL extract solution/1 mL DMEM) for 12 h. Scale bar, 50 *μ*m. (b) Comparison of the number of apoptotic cells with the TUNEL assay. (c) Annexin V/PI staining and flow cytometry analysis assessing apoptosis in GL261 cells. (d) The levels of PARP, active caspase-8, procaspase-8, and procaspase-9 in GL261 cells analyzed via Western blot. (e) Apoptosis analysis in GL261 glioma cells treated with cryoablated tumor extracts after caspase-8, caspase-9, or P2X7 was blocked. GL261 cells were treated with cryoablated tumor extracts (1 mL extract solution/mL DMEM), together with the inhibitors of caspase-8 (10 *μ*M IETD), caspase-9 (10 *μ*M LEDH), and P2X7 (10 *μ*M A740003), respectively, for 12 h. Compared with the control group, *P < 0.01.

We next investigated the effects of the extracts from glioma tissues after cryoablation on the GL261 glioma cells* in vitro*. To examine the effects of these extracts on the apoptosis in GL261 cells, TUNEL staining and flow cytometry analysis were conducted after these cells were treated with the cryoablated tumor extracts for 12 h. Extracts from sham-operated tumors were used as control. In TUNEL staining, cryoablated tumor extracts showed a strong effect in inducing apoptosis (apoptosis rate of 32% in the group treated with cryoablated tumor extracts versus 4% in the control group; *P* < 0.01) (Figures 2(a) and 2(b)). In line with the TUNEL staining, flow cytometry analysis showed that the cryoablated tumor extracts induced stronger apoptosis in GL261 cells, compared with the control group (*P* < 0.01) (Figure 2(c)). These data strongly suggest that the cryoablated tumor extracts have a strong potential in inducing apoptosis.

To determine the pathway through which the cryoablated tumor extracts induced apoptosis* in vitro*, Western blot was conducted to detect the expression levels of apoptotic proteins (caspase-8, caspase-9, and PARP) in GL261 cells treated with cryoablated tumor extracts for 12 h. Our results showed that, in the group treated with cryoablated tumor extracts, the protein levels of procaspase-8 were significantly decreased, with a strong increase in the expression level of its cleaved form (Figure 2(d)), indicating the extrinsic pathway. Although slight decreases in the expression levels of procaspase-9 and PARP were detected in the group treated with cryoablated tumor extracts (Figure 2(d)), this might be a consequence of the activation of caspase-8. These cells were further treated with the cryoablated tumor extracts, together with the inhibitors of caspase-8, caspase-9, and P2X7 ATP receptor, respectively, for 12 h. The TUNEL staining assay indicated that the blockages of P2X7 and especially caspase-8 could significantly inhibit the apoptotic process induced by the cryoablated tumor extracts in GL261 cells, while caspase-9 blockage did not affect the apoptosis-triggering effects of these extracts (Figure 2(e)). These results suggest that the cryoablated tumor extracts induce apoptosis in GL261 cells, mainly via the activation of caspase-8, which involves ATP as well.

### 3.4. Cryoablated Tumor Extracts Inhibit the Migration and Proliferation of GL261 Glioma Cells

**Figure 3:**
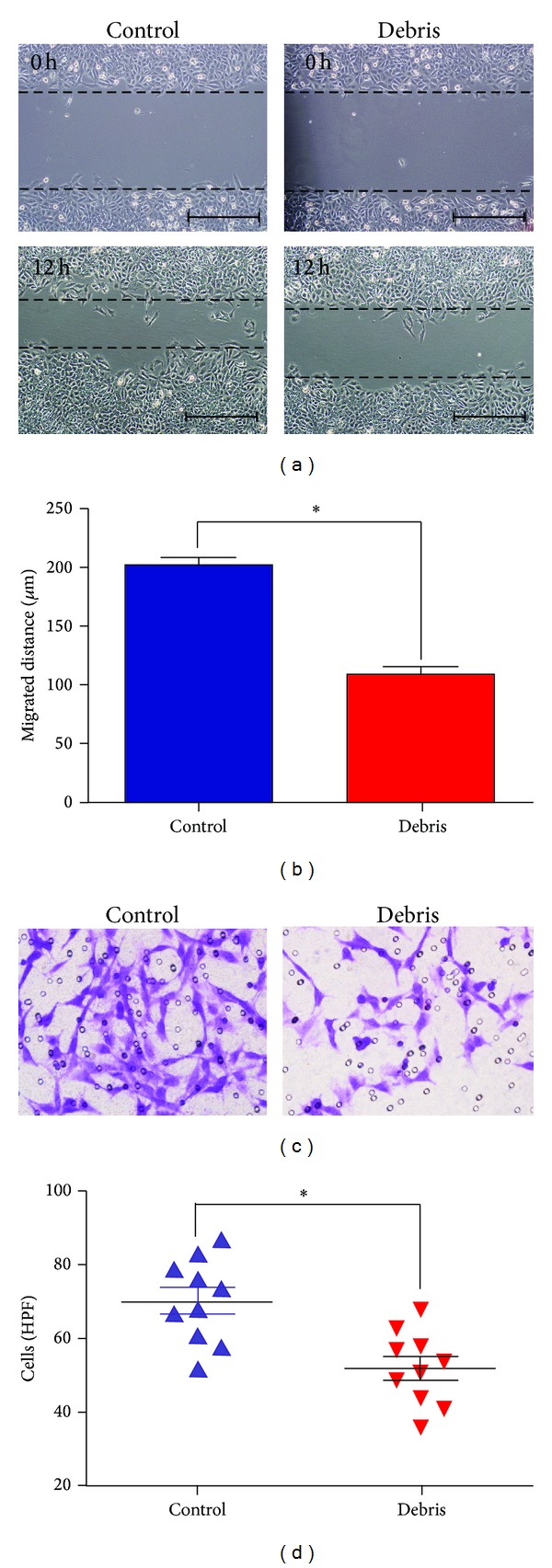
Cryoablated tumor extracts inhibit the migration and invasion of GL261 glioma cells. (a) Scratch assay detecting the migration of GL261 cells treated with cryoablated tumor extracts for 12 h. Scale bar, 50 *μ*m. (b) Comparison of the migrated distance of the GL261 cells in the cryoablated tumor extract group and the control group. (c) Transwell assay evaluating the invasion of the GL261 glioma cells. (d) Comparison of the number of invaded tumor cells in the cryoablated tumor extract group and the control group. Compared with the control group, *P < 0.01.

To find out if the cryoablated tumor extracts could suppress the migration and invasion of the tumor cells, the scratch assay was conducted. We found that the migrated distance of GL261 cells treated with cryoablated tumor extracts was about half of that of cells treated control extracts (Figures 3(a) and 3(b)), with an inhibitory rate of 47%. Furthermore, the transwell assay indicated that cryoablated tumor extracts significantly decreased the number of invasive GL261 cells (*P* < 0.01) (Figures 3(c) and 3(d)). These data suggest that cryoablated tumor extracts play an inhibitory role in migration and invasion of GL261 cells.

**Figure 4:**
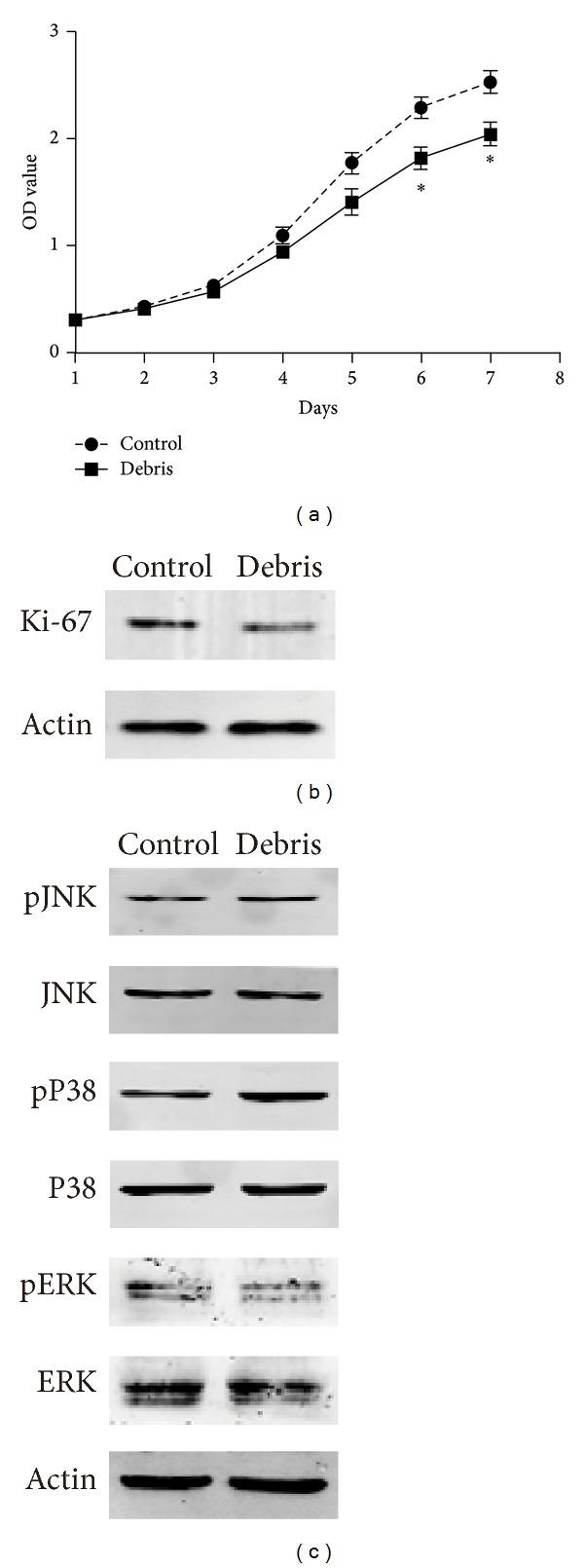
Cryoablated tumor extracts inhibit the proliferation of GL261 glioma cells. (a) Growth status of the GL261 cells in the group treated with cryoablated tumor extracts and the control group on days 1–7 analyzed via CCK-8 assay. Compared with the control group, *P < 0.01. (b) Levels of Ki-67 in the GL261 cells detected by Western blot. (c) Levels of phosphorylation of P38, ERK, and JNK in the GL261 cells analyzed by Western blot. GL261 cells were treated with cryoablated tumor extracts (1 mL extract solution/1 mL DMEM) for 12 h.

We found that GL261 cells treated with cryoablated tumor extracts took more time to reach the same confluence with the control cells (data not shown). So we conducted CCK-8 to test the impact of the cryoablated tumor extracts on the proliferation of GL261 cells. In this assay, cells treated with cryoablated tumor extracts showed a lower growth rate compared with the control group (Figure 4(a)). In line with this, Western blot analysis indicated that the expression level of Ki-67, a well-known marker for tumor cell proliferation, was lower in the group treated with cryoablated tumor extracts than in the control group (*P* < 0.05) (Figure 4(b)), indicating the inhibitory effect of cryoablated tumor extracts on tumor cell proliferation.

### 3.5. Cryoablated Tumor Extracts Inhibit the ERK1/2 Pathway and Activate the P38 Pathway in GL261 Glioma Cells

Enhanced invasion and proliferation of tumor cells have always been connected with the activation of MAPKs pathways, including P38, ERK1/2, and JNK [15, 16]. Additionally, P38 pathway is also closely related to cellular apoptosis [17]. To test whether the reduced invasion and cell viability induced by cryoablated tumor extracts were companied with the alterations in MAPKs pathways, Western blot was conducted to detect the phosphorylation levels of P38, ERK1/2, and JNK in GL261 glioma cells. In this assay, there were no significant differences in phosphorylation levels of JNK between the group treated with cryoablated tumor extracts and the control group. However, the phosphorylation level of ERK1/2 was lower, and the phosphorylation level of P38 was higher, in the cryoablated tumor extract-treated group, compared with the control group (Figure 4(c)). These data indicate that the cell migration and proliferation inhibiting effects of cryoablated tumor extracts might be related to the inhibition of ERK1/2 pathway and the activation of P38 pathway.

## 4. Discussion

Increasing evidence shows that cryoablation causes apoptosis [18, 19], but the detailed mechanism is not fully understood. Apoptosis, or programmed cell death, is characterized by cell shrinkage, membrane blebbing, chromatin condensation, genomic fragmentation, and caspase activation [20]. Numerous cellular factors have been associated with the apoptotic signaling pathway. There are two commonly recognized apoptotic signal-transduction pathways: the receptor-mediated (extrinsic) pathway and the mitochondrial-mediated (intrinsic) pathway. The extrinsic pathway is characterized by activation of caspase-8, which is induced by death receptor ligands, loss of growth factor signaling, and alterations in cell attachment signaling. The intrinsic pathway is characterized by the “opening” of the mitochondrial permeability transition pore, release of cytochrome-c, proteolytic cleavage of poly (ADP-ribose) polymerase (PARP), and activation of caspase-9. Studies of cryoablation on prostate cancer cells* in vitro* suggest that low temperature (−30°C~−15°C) leads to cell death mainly via mitochondrial-mediated apoptosis [21, 22]. In our study, we detected two distinct waves of apoptosis in glioma tissues following cryoablation, that is, the rapid-onset apoptosis and the delayed apoptosis. These two waves of apoptosis occurred at different time points and different locations, via different pathways. The rapid-onset apoptosis was mainly induced by low temperature via the intrinsic pathway, while the delayed apoptosis was mainly triggered via the extrinsic pathway. There are two possible reasons for the delayed apoptosis. It might be caused by the hypoxia induced by microcirculatory failure. On the other hand, the cellular contents released from the damaged cells would also be responsible. To exclude possibility of hypoxia, we conducted the cell experiments under aerobic conditions. The results from the TNUEL and flow cytometry analysis demonstrated the proapoptotic ability of the cryoablated tumor extracts under these conditions (data not shown). Moreover, cryoablated tumor extract-induced GL261 cell apoptosis was mainly activated via the extrinsic apoptotic pathway. Slow penetration and/or diffusion of the released cellular contents may explain why the second wave of apoptosis in the glioma tissues after cryoablation occurred in the S3 area and why it was delayed.

In addition to proapoptotic ability of cryoablated tumor extracts, we also found that they have effects of antiproliferation and anti-invasion on tumor cells. Our results further suggested that these inhibitory effects were related to the inhibition of ERK1/2 pathway and the activation of P38 pathway. Up to now, there are few studies focused on the compositions and functions of the cryoablated tumor extracts or the cellular contents released from cryodamaged cells. Therefore, further studies are needed to explore the precise compositions in the extracts that play important roles in inhibiting cell proliferation and invasion.

In conclusion, we found two successive waves of apoptosis in the glioma tumors following cryoablation: the rapid-onset apoptosis, directly caused by freezing via the intrinsic pathway, and the delayed apoptosis induced by the released cellular contents via the extrinsic pathway. Besides, cryoablated tumor extracts showed* in vitro* inhibiting effects on cell proliferation and migration, which might contribute to the further understanding of the internal mechanism of cryoablation. Therefore, since glioma cells surviving under sublethal temperature would subsequently undergo intrinsic or extrinsic apoptosis, augmenting the induction of apoptosis or enhancing the cryosensitization of tumor cells by coupling cryoablation with specific chemotherapy might effectively increase the efficiency of this therapeutic treatment.

## Abbreviations

ATP: Adenosine triphosphate
CCK-8: Cell counting kit-8
DMEM: Dulbecco's modified Eagle's medium
FBS: Fetal bovine serum
MAPKs: Mitogen activated protein kinase pathways
PARP: Poly (ADP-ribose) polymerase
TUNEL: Terminal deoxynucleotidyl transferase dUTP nick end labeling.
